# Fast Evolving Glioblastoma in a Pregnant Woman: Diagnostic and Therapeutic Challenges

**DOI:** 10.3390/diagnostics15151836

**Published:** 2025-07-22

**Authors:** Ivan Bogdanovic, Rosanda Ilic, Aleksandar Kostic, Aleksandar Miljkovic, Filip Milisavljevic, Marija M. Janjic, Ivana M. Bjelobaba, Danijela Savic, Vladimir Bascarevic

**Affiliations:** 1 University Clinical Center of Serbia, Clinic for Neurosurgery, 11000 Belgrade, Serbia; ivanbg83@gmail.com (I.B.); rosandailic@gmail.com (R.I.); aleksandar00k@gmail.com (A.K.); aleksandar.miljkovic89@hotmail.com (A.M.); milisavljevic93@gmail.com (F.M.); 2 School of Medicine, University of Belgrade, 11000 Belgrade, Serbia; 3 Institute for Biological Research “Siniša Stanković”-National Institute of Republic of Serbia, University of Belgrade, 11000 Belgrade, Serbia; marija.janjic@ibiss.bg.ac.rs (M.M.J.); ivana.bjelobaba@ibiss.bg.ac.rs (I.M.B.); danisto@ibiss.bg.ac.rs (D.S.)

**Keywords:** pregnancy, brain cancer, magnetic resonance spectroscopy, glioblastoma therapy, maternal–fetal outcome

## Abstract

**Background and Clinical Significance:** Gliomas diagnosed during pregnancy are rare, and there are no established guidelines for their management. Effective treatment requires a multidisciplinary approach to balance maternal health and pregnancy preservation. **Case Presentation:** We here present a case of rapidly progressing glioma in a 33-year-old pregnant woman. The patient initially presented with a generalized tonic–clonic seizure at 21 weeks’ gestation. Imaging revealed a tumor in the right cerebral lobe, involving both cortical and subcortical structures, while magnetic resonance spectroscopy suggested a low-grade glioma. The patient remained clinically stable for two months but then developed severe headaches; MRI showed a worsening mass effect. At 34 weeks’ gestation, an emergency and premature caesarean section was performed under general anesthesia. The patient then underwent a craniotomy for maximal tumor resection, which was histologically and molecularly diagnosed as IDH wild-type glioblastoma (GB). Using qPCR, we found that the GB tissue showed upregulated expression of genes involved in cell structure (*GFAP*, *VIM*) and immune response (*SSP1*, *TSPO*), as well as increased expression of genes related to potential hormone response (*AR*, *CYP19A1*, *ESR1*, *GPER1*). After surgery, the patient showed resistance to Stupp protocol therapy, which was substituted with lomustine and bevacizumab combination therapy. **Conclusions:** This case illustrates that glioma may progress rapidly during pregnancy, but a favorable obstetric outcome is achievable. Management of similar cases should respect both the need for timely treatment and the patient’s informed decision.

## 1. Introduction

The diagnosis of glioma tumors during pregnancy is rare, and there are no specific guidelines for its treatment. Instead, clinicians rely on limited case reports or small retrospective studies to guide their clinical approach and determine the optimal timing for tumor surveillance and treatment [1,2,3,4,5]. GB represents 14% of all primary brain tumors, but it is the most prevalent malignant form of both primary brain tumors and gliomas, comprising 51% and 60% of these categories, respectively [6]. The incidence of GB varies depending on the geographical region and is between 0.59 and 3.69 per 100,000 people, with men being affected more frequently (1.6 times higher incidence) [7]. Since 2005, the Stupp protocol is the standard of care for patients with newly diagnosed GB [8]. This protocol usually involves surgical resection of the tumor as far as possible, followed by tumor-focused radiotherapy and chemotherapy with temozolomide, which is administered daily during radiotherapy and continued over several cycles after radiotherapy has finished. Even under this protocol the median overall survival for GB patients is generally poor, typically around 14–16 months [7]. Although attempts have been made to develop new therapeutic approaches such as immunotherapies and vaccines, there have been no significant advances that would substantially improve the life expectancy of GB patients [9].

The coexistence of pregnancy and glioma requires a multidisciplinary medical team to create an optimal treatment plan for the management of the pregnancy and eradication of the tumor, tailored to the patient’s priorities and preferences. This plan may aim to deliver a healthy baby, even if it means delaying glioma treatment until after pregnancy, or it may prioritize aggressive glioma treatment, possibly at the expense of fetal survival [10]. To the best of our knowledge, the last study investigating the incidence of primary brain tumors in pregnant versus non-pregnant women was published in 1987 [11], reporting a similar incidence of gliomas. Several studies have investigated the effects of pregnancy on WHO grade II and III gliomas, as well as GB and vice versa, examining both the cases where the first symptoms of the tumor occurred during pregnancy, as well as those in which the tumor was diagnosed before pregnancy [4,5,10,12,13]. Overall, regardless of the timing of tumor diagnosis these studies came to the conclusion that pregnancy is positively correlated with clinical deterioration. Tumor progression during pregnancy was more likely to be associated with gliomas that had a high grade of malignancy, immunonegative expression of alpha-internexin, or immunopositive expression of p53 [4]. Furthermore, tumor growth progression was quantitatively confirmed by an increased rate of diametric expansion observed in consecutive magnetic resonance images of pregnant patients [13]. In the study of Peters et al., it was noted that 83% of gliomas diagnosed during pregnancy were discovered in the second or third trimesters, with seizures being the presenting symptom in 68% of cases [4].

Hormonal and immunological changes during pregnancy can influence the development and progression of tumors. Certain pregnancy-related hormones have been associated with modulation of GB behavior. Interestingly, a common feature of pregnancy and GB is the production of human chorionic gonadotropin (hCG). GB cells have been shown in vitro to secrete hCG-β, which exerts an autocrine effect by scavenging reactive oxygen species and promoting cell survival through anti-apoptotic mechanisms [14]. Notably, hCG levels rise sharply during the first trimester of pregnancy, a period characterized by extensive hormonal modulation and immune adaptation. This suggests that in cases where pregnancy and GB coexist, hCG may contribute not only to fetal–maternal immune tolerance but also to GB cell survival by regulating redox homeostasis.

All of these reports clearly indicate that pregnant patients with a new or prior diagnosis of glioma should be informed that pregnancy may significantly increase the risk of tumor progression and that both untreated glioma and current treatment protocols carry potential risks to fetal development. The available literature—primarily retrospective studies with small cohorts—suggest that favorable pregnancy outcomes are possible with careful management and close monitoring, and that healthy babies were born in the majority of reported cases [4,10,15]. However, it is important to note that data are limited, and, to the best of our knowledge, long-term follow-up of children born under these circumstances has not been reported.

Here we present a newly diagnosed glioma in a pregnant woman at the end of the second trimester whose symptoms worsened in 32nd week of gestation, leading to premature caesarean section and surgical removal of the tumor, which was confirmed as a WHO grade IV IDH wild-type GB. To explore the molecular profile of the tumor with a focus on genes related to sex hormone pathways, we assessed the expression of *AR*, *ESR1*, *GPER1*, and *PGR*, along with *CYP19A1*, which encodes aromatase, an enzyme involved in the conversion of androgens to estrogens. In addition, we assessed the expression of genes associated with tumor aggressiveness and possible therapy resistance (*GFAP*, *VIM*, *SPP1*, and *TSPO*).

## 2. Case Description and Discussion

### 2.1. Anamnesis

**Figure 1:**
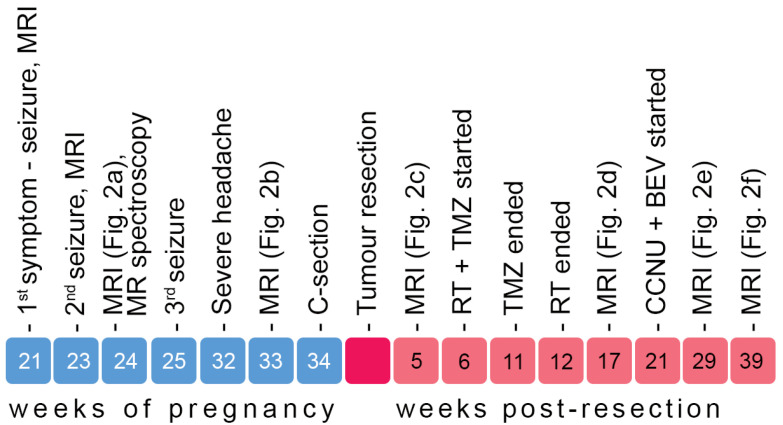
Timeline of major clinical events, from symptom onset during pregnancy to 9 months post-resection. The figure shows timing of key MRI scans (corresponding to Figure 2a–f), seizures, delivery (C-section), surgery, and the start and end of adjuvant treatments. RT—radiotherapy; TMZ –temozolomide; CCNU—chloroethyl-cyclohexyl-nitrosourea (lomustine); BEV—bevacizumab.

A 33-year-old woman with a predisposition to thrombophilia due to genetic mutations became pregnant after the second attempt at IVF. She was admitted to the clinic after the first neurological symptom: a generalized tonic–clonic seizure at the 21st week of pregnancy. The patient was awake and responsive, without signs of neurological deficits and the electroencephalogram was in the physiological range. The patient had no personal or family history of neurological disease or brain tumors. Of the pre-existing conditions, only mild myopia was reported. Apart from subcutaneous enoxaparin sodium (40 mg/day) and vaginal progesterone (600 mg/day) from IVF onwards, no long-term medication was reported. The further course of her condition is outlined in Figure 1.

**Figure 2:**
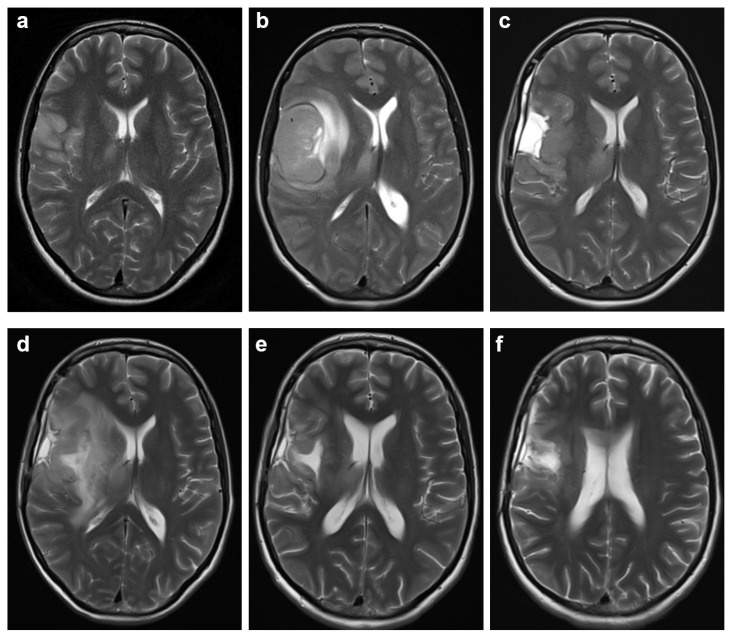
Sequential T2-weighted axial brain MRIs over a 12-month period, corresponding to the timeline shown in Figure 1. (**a**) MRI at 24 weeks of pregnancy showing a hyperintense lesion in the right frontal lobe. (**b**) MRI at 33 weeks of pregnancy showing significant enlargement of the lesion. (**c**) Postoperative MRI five weeks after tumor resection. (**d**) MRI four months after resection showing tumor recurrence and progression despite surgery and radio-chemotherapy (RT + TMZ). (**e**) MRI seven months after resection, following two cycles of lomustine and four administrations of bevacizumab, demonstrating radiological regression. (**f**) MRI nine months after resection showing further tumor regression following three cycles of lomustine and nine administrations of bevacizumab, along with six doses of personalized anticancer vaccine administered abroad.

### 2.2. Symptom Development, Imaging, and Surgical Intervention

Although antiepileptic drugs carry a risk of congenital malformations, untreated seizures during pregnancy pose a significantly higher risk for mother and fetus [16]. Treatment is therefore essential as the benefits are generally considered to outweigh the potential risks. The patient was started on a monotherapy with levetiracetam, a first-line treatment considered safe for both general and tumor-induced epilepsy during pregnancy [17]. Antiepileptic drugs, including levetiracetam, are associated with an increased risk of neural tube defects in the developing fetus, and it is important to note that the patient was already supplementing folic acid [17,18]. The first MRI, performed after the first seizure, showed a hyperintense T2-weighted and FLAIR lesion involving the right frontal cortex, insula, with additional involvement of the temporal cortex, the head of the caudate nucleus, the body of the corpus callosum, and the right thalamus. Due to concerns regarding pregnancy and fetal safety, MRI was performed without contrast, as this is the technique of choice for monitoring a pregnant patient diagnosed with a brain tumor [15]. Therefore, the lesion appeared isointense on T1-weighted imaging. No significant changes were detected on the second and the third MRI examination (T2-weighted MRI shown in Figure 2a) performed at 24 weeks’ gestation. Due to two additional seizures that occurred up to the 25th week of pregnancy (manifested by stiffness of the lower jaw, tingling of the tongue and left arm), the initial dose of levetiracetam was gradually increased from 1000 mg to 3000 mg daily. Enoxaparin sodium was continued.

The third MRI examination (at 24 weeks of pregnancy, Figure 1 and Figure 2a) was accompanied by an analysis of the chemical composition of the tumor with a single and 2D multivoxel MR spectroscopy. This revealed a slight increase in the choline/creatine ratio (Cho/Cr = 1.49–1.53—the highest registered value), along with a decrease in the N-acetylaspartate/creatine (NAA/Cr = 0.92–1.27) and choline/N-acetylaspartate ratios (Cho/NAA = 1.19–1.62), while myoinositol levels remained within the reference range (mI/Cr = 0.61). Although these metabolic changes suggest altered cell metabolism or membrane turnover, some loss of neurons and an unaffected glial cell component, their extent is consistent with a low-grade glioma. However, we acknowledge that MR spectroscopy alone cannot reliably exclude the presence of high-grade glioma, including molecular glioblastoma, and it is not routinely used in practice in Serbia to define tumor grade. In this particular case, due to the patient’s pregnancy and the decision to avoid gadolinium-based contrast, MR spectroscopy was used as an adjunct imaging modality. The patient’s young age (33 years) and the fact that her first and only symptom was a generalized seizure—commonly associated with lower-grade gliomas—were additional factors considered when forming the initial management plan.

In pregnant patients with newly diagnosed glioma, surgery in the second trimester may be recommended if necessary, as surgery in the first trimester poses a high risk to fetal survival, while surgery in the third trimester increases the risk of significant intraoperative bleeding [10,16]. Based on the imaging results and the timing of patient’s initial neurosurgical consultation—towards the end of the second trimester—emergency neurosurgical intervention was not indicated. Instead, intensive antenatal and neurological monitoring was recommended along with symptomatic treatment, and definitive oncological treatment was postponed until after delivery.

Regarding the etiology of the glioma, which remains unknown in most cases, the patient stated that she had not been exposed to high doses of ionizing radiation in the past, the only external risk factor known to be associated with this type of brain tumor [19]. She also denied any previous exposure to cranial diagnostic radiation (computed tomography), which, in childhood or before the age of 22, is a factor that could increase the risk of developing glioma [20].

The next symptom developed at 32 weeks’ gestation and presented as a tension-type headache during a routine visit to the obstetric clinic. The headache was characterized by generalized pain that did not respond to paracetamol but was not accompanied by other symptoms like nausea or vomiting. The deterioration occurred between the 24th and 32nd week of gestation, coinciding with the period when pregnancy-related hemodynamic changes reach their peak [21]. During this period, increased blood volume and cardiac output together with decreased systemic vascular resistance support the metabolic demands of pregnancy and fetal development. However, these changes also lead to an increase in maternal cerebral blood flow, which may promote tumor vascularization, the formation of peritumoral edema, and the progression of the tumor itself [21,22]. Moreover, the production of certain growth factors by the placenta, including placental growth factor, vascular endothelial growth factor, and insulin-like growth factors, peaks in the second trimester and remains elevated throughout the duration of pregnancy. These factors are known to promote angiogenesis, glioma cell migration, and tumor growth in GB. Therefore, they also may play a role in the progression of GB during pregnancy.

The MRI scan followed at the 33rd week of pregnancy (Figure 2b), revealing the progressive mass effect. The lesion had enlarged significantly, resulting in perilesional edema and compressive effects on the ventricular system with a pronounced shift to the left. This was accompanied by subfalcine herniation, dilatation of the left lateral ventricle, initial transependymal cerebrospinal fluid resorption, and diffuse cerebral edema. An emergency surgical intervention was scheduled following the caesarean section.

Corticosteroids were administered to promote lung maturity in the fetus, and the baby was delivered by caesarean section under general anesthetic at 34 weeks’ gestation. The delivery was uneventful; the baby cried spontaneously and achieved an Apgar score of 9 at both one and ten minutes after birth. Five days after delivery and three months after the initial symptom, the patient underwent a craniotomy for maximal surgical resection of the tumor. Total resection was not possible due to the infiltration of deep brain structures. The operation was successful, and the postoperative course was uneventful, with stable vital signs and no complications.

### 2.3. Pathology and Molecular Analysis

**Figure 3:**
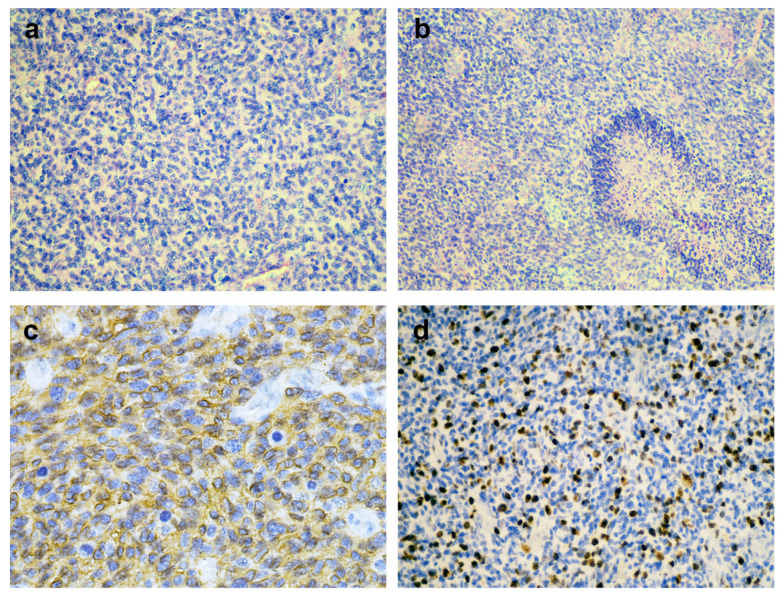
Hematoxylin and eosin staining (**a**,**b**) and immunohistochemistry (**c**,**d**) of GB, IDH-wild type, CNS WHO grade 4. (**a**) Hypercellular sheets of anaplastic glial cells. (**b**) Palisading necrosis and microvascular proliferation with endothelial hyperplasia. (**c**) GFAP immunoexpression by tumor cells. (**d**) High Ki-67 proliferation index.

Histopathological analysis of the tumor showed poorly differentiated, primitive-looking cells, significant microvascular proliferation, abnormal mitotic activity, and moderate necrosis (Figure 3a,b). Immunohistochemistry revealed positive staining for GFAP (Figure 3c), Olig2, Vimentin, MAP2, p53, and retained H3K27me3. The tumor cells were negative for IDH-1 R132H, CDKN2A, and synaptophysin. In addition, ATRX expression was preserved and the Ki-67 proliferation index was 30% (Figure 3d). These immunohistochemical results are summarized in Supplementary Table S1 to aid interpretation.

PCR analysis indicated the absence of mutations in codon 132 of the IDH1 gene and in codons 140 and 172 of the IDH2 gene. Fluorescence in situ hybridization with 1p36/1q25 and 19q13/19p13 probes revealed a loss of heterozygosity at 19q13 in the majority of neoplastic cells. The full molecular profile, including its diagnostic relevance, is also presented in Supplementary Table S1. Based on these results and according to the WHO 2021 classification for CNS tumors, a diagnosis of IDH wild-type GB, grade IV, was established.

**Figure 4:**
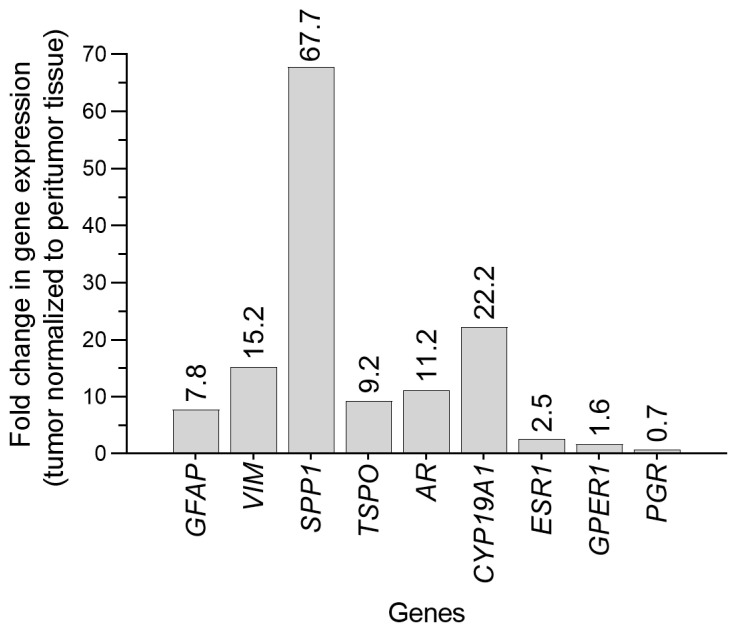
Relative expression of genes associated with cell structure, immune response, and hormone signaling in GB. Expression levels of target genes (*GFAP*, *VIM*, *SPP1*, *TSPO*, *AR, CYP19A1*, *ESR1*, *GPER1,* and *PGR*) in tumor tissue are shown relative to the expression of the corresponding gene in peritumor tissue. Tissue samples were collected during surgery and preserved in RNAlater^®^ RNA Stabilization Solution. Total RNA was extracted with TRIzol reagent. After determining RNA concentrations, reverse transcription was carried out with a High Capacity cDNA Reverse Transcription Kit; qPCR analysis was performed using the QuantStudio™ 3 Real-Time PCR System with SYBR™ Green reagent. All chemicals and equipment were sourced from Thermo Fisher Scientific, Waltham, MA, USA and full methodological details are provided in the Supplementary Materials. The expression levels of target genes were quantified by comparative 2^−ΔCt^ method, using the hypoxanthine phosphoribosyltransferase gene (*HPRT1*) as a housekeeping gene. Primer sequences are listed in Supplementary Table S2.

Gene expression in signaling pathways related to hormonal regulation was analyzed using quantitative real-time PCR (qPCR) on both the tumor and surrounding tissue, aiming to explore the molecular phenotype of the tumor concurrent with pregnancy (Figure 4). The surrounding tissue sample is referred to here as peritumor tissue. To confirm the distinction between tumor and peritumor tissue, we also assessed the expression of genes involved in cell structure and immune response that are typically upregulated in GB cells, such as *GFAP*, *VIM*, *SPP1*, and *TSPO*. The qPCR methodology, including a list of primer sequences used (Supplementary Table S2), is detailed in the Supplementary Materials.

GFAP, an intermediate filament and structural component of mature astrocytes, is highly expressed in tumors of astrocytic origin, including GB, and is frequently used as an immunohistochemical marker for these tumors [23]. Interestingly, GFAP can even be detected in the plasma of GB patients [23]. However, its expression has been reported to correlate both positively and negatively with tumor malignancy [24]. In our case, GFAP mRNA levels were 8-fold higher in tumor compared to peritumor tissue (Figure 4). Similarly, the expression of another intermediate filament *VIM* was 15- fold higher in the tumor sample.

Our results also show *SPP1* to be highly induced in GB tissue, with 68-fold higher expression than in peritumor tissue (Figure 4). Osteopontin, a glycophoshoprotein encoded by the *SPP1* gene, is a potent chemokine for macrophages and plays a critical role in GB malignancy, recruiting M2 macrophages into the tumor, thus creating a pro-tumor immune microenvironment [25]. Hyper-phosphorylation of osteopontin has also been shown to be associated with poorer survival only in female GB patients, suggesting potential sex-specific effects [26].

Finally, both VIM and osteopontin are associated with enhanced tumor cell migration, invasion, and poor prognosis in GB, with osteopontin additionally contributing to radioresistance [27,28,29,30].

We also found that *TSPO* expression was upregulated in tumor tissue (Figure 4). *TSPO* encodes a mitochondrial membrane protein involved in cellular processes such as proliferation, apoptosis, and immune signaling, and is overexpressed in gliomas, including GB [31,32].

Taken together, the ≥9-fold increase in *VIM*, *SPP1*, and *TSPO* expression in tumor compared to peritumor tissue reflects molecular features typically associated with aggressive GB behavior. However, as our analysis was limited to mRNA expression, further studies are needed to confirm whether these changes translate to increased protein levels and functional effects.

Although GBs are not typically classified as hormone-sensitive cancers, significant sex-related differences have been observed, with males exhibiting a higher incidence and worse prognosis [33]. These epidemiological patterns have prompted speculation that sex hormones may influence GB biology, with androgens being linked to tumor-promoting effects and estrogens to potentially protective effects [33].

Our findings show 11-fold higher AR mRNA levels in GB tissue versus peritumor tissue (Figure 4), which aligns with previous studies reporting increased *AR* gene expression (at least 2.5-fold) and elevated protein levels in GB biopsies relative to normal brain tissue [34,35,36]. Higher *AR* expression is positively correlated with glioma grade, being most pronounced in GB, and is associated with poorer survival across all glioma subtypes [35,37]. Activation of AR in the context of GB enhances the malignant properties of tumor cells, promotes an immunosuppressive tumor environment and contributes to radioresistance [36,38]. Notably, androgen levels rise significantly during pregnancy, peaking in the third trimester [39], raising the possibility that elevated androgens could activate AR-mediated signaling in GB cells during this time. While we acknowledge the limitations of drawing functional conclusions from gene expression data alone, this temporal overlap may warrant further investigation.

In contrast, the role of estrogens and their receptors in GB appears more complex and remains controversial. Studies have reported both increased and decreased expression of ERα and ERβ in GB, with conflicting associations with tumor grade and clinical outcomes [40,41]. These receptors have been linked to both poor prognosis and longer survival [40,42,43,44], highlighting the nuanced and context-dependent nature of estrogen signaling. Functional data suggest that estradiol may promote tumor growth via ERα, while ERβ may have protective effects [45,46]. We also observed a 2.5-fold increase in *ESR1* expression in tumor tissue (Figure 4), consistent with previous findings [40], whereas *ESR2* levels were very low in both tumor and peritumor samples (Ct > 30). These results contribute to ongoing debates about the role of estrogen receptors in GB, but definitive conclusions cannot be drawn from mRNA expression alone.

Similarly, the role of aromatase, which converts androgens to estrogens, is unclear. While some studies associate its expression with poor survival [43], others suggest a favorable prognosis [44]. In our study, we observed a 22-fold increase in *CYP19A1* expression in tumor tissue (Figure 4), indicating possible aromatase activity in the tumor microenvironment. Ongoing clinical trial investigating the aromatase inhibitor letrozole may help define the therapeutic potential of this pathway [47].

Estradiol may also act through membrane-bound GPERs, which mediate rapid signaling responses. One study reported that high *GPER1* expression was associated with favorable outcomes in female GB patients [48]. In our case, *GPER1* was modestly increased in tumor tissue (Figure 4).

Finally, we observed a slight decrease in *PGR* expression in GB tissue compared to peritumor tissue (Figure 4). Progesterone has been shown to exert complex, dose-dependent effects on GB cells, with low concentrations promoting and higher concentrations suppressing tumor growth [49]. Although progesterone levels rise substantially during pregnancy, glioma progression is often accelerated in pregnant patients [4,5,10,12,13]. Reduced *PGR* expression may suggest limited responsiveness to potential anti-tumor effects of progesterone, though this association remains to be clarified.

We would like to emphasize that our primary goal was to present gene expression patterns in the rare clinical context of the coexistence of GB and pregnancy. While the analyzed genes are known to be associated with the pathogenesis of GB, we acknowledge that no definitive conclusions can be drawn from this single case or from gene expression data alone. Due to the limited amount of peritumor tissue, protein-level validation was not feasible, which restricts functional interpretation of the observed mRNA changes. However, we note that the radiological progression of the tumor documented on MRI between 24 and 33 weeks of gestation coincided with the period when pregnancy-related hemodynamic changes and growth factor production peak, suggesting a possible influence of the pregnancy environment on tumor behavior. This temporal association is presented as a hypothesis-generating observation that warrants further investigation in larger, systematically analyzed cohorts.

### 2.4. Postoperative Course

Five weeks after the tumor resection, the MRI follow-up (Figure 2c) confirmed that the surgical resection was sufficient so that the patient could begin fractionated radiotherapy (60 Gy, 2 Gy/fraction) along with simultaneous chemotherapy with temozolomide (120 mg/day). The radiotherapy lasted six weeks; the chemotherapy was discontinued after five weeks due to grade 1 thrombocytopenia. One month after completion of radiotherapy, the patient developed severe right-sided headaches accompanied by nausea and vomiting. T2-weighted (Figure 2d) and FLAIR MRI showed a hyperintense lesion together with a centrally located FLAIR hypointensity, indicating recurrence of the tumor with significant growth, infiltration of deep brain structures, and necrosis. Due to the spread of the tumor into deep brain structures, surgical reoperation was not indicated, and the patient was started on anti-edema therapy.

As the patient showed early resistance to the Stupp protocol (Figure 2d), second-line chemotherapy was initiated, consisting of lomustine (80 mg orally every six weeks) and off-label bevacizumab (10 mg/kg intravenously every two weeks). A follow-up MRI (Figure 2e) after two cycles of lomustine and four administrations of bevacizumab (performed seven months after tumor resection) demonstrated radiological regression compared to the prior scan (Figure 2d). The hyperintense zones in the surgical area and surrounding structures appeared less extensive, with no signs of mass effect or midline shift. The findings were interpreted as post-therapeutic changes and consistent with a positive response to treatment. The patient reported a subjective improvement and no new neurological symptoms.

After this, the patient independently decided to initiate an immunotherapy with a personalized anticancer vaccine. As this treatment was conducted outside our institution, we do not have access to additional clinical details. A subsequent MRI, performed after three cycles of lomustine and nine bevacizumab administrations along with six out of fourteen anticancer vaccine doses, confirmed further regression (Figure 2f). In this MRI, performed nine months after resection, the surgical cavity appeared slightly reduced in size, and the surrounding hyperintensity further decreased. When compared to the previous MRIs, the current findings indicate sustained radiological improvement and ongoing tumor regression. At the time of the most recent imaging, the patient remained stable, functional, and without significant neurological deficits. Therapy with lomustine and bevacizumab is ongoing.

## 3. Conclusions

This case illustrates the rapid progression of a GB in a patient diagnosed during pregnancy after IVF treatment. Classical and modern MR techniques initially suggested a low-grade glioma, highlighting the limitations of imaging and spectroscopy in accurately predicting glioma grade and progression. In addition, early-stage GBs may have relatively low metabolic activity, as reflected by Cho/Cr and Cho/NAA ratios, before more pronounced features such as necrosis, vascularity, and rapid cell division appear. The literature reports have shown that, even in the absence of overt radiological or histological features of high-grade glioma, a tumor may carry the molecular signature of glioblastoma and later evolve into its full pathological and neuroradiological phenotype [50].

According to the WHO 2021 classification, the presence of specific molecular alterations—such as the simultaneous gain of chromosome 7 and loss of chromosome 10, TERT promoter mutation, or EGFR amplification—allows for a diagnosis of molecular glioblastoma (IDH-wild type, WHO grade 4), even when histological features appear low-grade [51]. Although full implementation of the WHO 2021 classification remains limited in our setting due to the unavailability of next-generation sequencing and methylation profiling, it is reasonable to assume that the glioma in this case was IDH-wild type from the outset and underwent rapid progression from a low- to high-grade phenotype.

Our case underscores the potential of significant tumor progression over a short interval, despite a stable clinical presentation. Notably, this progression coincided with the pregnancy period characterized by hormonal and hemodynamic changes as well as placental production of growth factors, which might have contributed to tumor growth.

In conclusion, this case supports previous findings that glioma diagnosed during pregnancy may progress rapidly, yet a favorable obstetric outcome is still possible if the patient chooses to continue the pregnancy. Clinical management should therefore aim to carefully balance the need for timely tumor treatment with respect for the patient’s informed consent decision and the potential impact of medical interventions on fetal development.

## Data Availability

The data presented in this study are available on request from the corresponding author but will not be made publicly available to protect the privacy of patient.

## Supplementary Materials

The following supporting information can be downloaded at: https://www.mdpi.com/article/10.3390/diagnostics15151836/s1, Table S1: Summary of the immunohistochemical markers and molecular tests performed on the tumor tissue, their results, and their specific relevance for the diagnostic classification according to the 2021 WHO classification of central nervous system tumors. The findings support the diagnosis of IDH-wildtype glioblastoma, WHO grade IV; Table S2: Primer sequences used for qPCR analysis.

## Abbreviations

The following abbreviations are used in this manuscript:

AR	Androgen receptor
ATRX	Alpha-thalassemia/mental retardation, X-linked
CHT	Chemotherapy
CNS	Central nervous system
CYP19A1	Aromatase
ESR1	Estrogen receptor α
FLAIR	Fluid-attenuated inversion recovery
GB	Glioblastoma
GFAP	Glial fibrillary acidic protein
GPER	G protein-coupled estrogen receptor
hCG	Human chorionic gonadotropin
IDH	Isocitrate dehydrogenase
IVF	In vitro fertilization
Ki-67	Marker of proliferation Kiel 67
MAP2	Microtubule-associated protein 2
MRI	Magnetic resonance imaging
Olig2	Oligodendrocyte lineage transcription factor 2
p53	Tumor protein p53
PCR	Polymerase chain reaction
PGR	Progesterone receptor
RT	Radiotherapy
SSP1	Osteopontin
TSPO	Translocator protein
VIM	Vimentin
H3K27me3	Histone 3 Lys 27 trimethylation
CDKN2A	Cyclin-dependent kinase inhibitor 2A
