# Supplementary material for: Fast Evolving Glioblastoma in a Pregnant Woman: Diagnostic and Therapeutic Challenges
Source: Diagnostics (Basel). 2025 Jul 22;15(15):1836. doi: 10.3390/diagnostics15151836 (PMC12345949; doi:10.3390/diagnostics15151836)
Supplement: Supplementary file 1 [file diagnostics-15-01836-s001.zip › diagnostics-3675942-supplementary.pdf]

Supplementary Table S1. Summary of the immunohistochemical markers and molecular tests performed on the tumor tissue, their results, and their specific relevance for the diagnostic classification according to the 2021 WHO classification of central nervous system tumors. The findings support the diagnosis of IDH-wildtype glioblastoma, WHO grade IV.

Marker/Test	Result	Diagnostic[1] Relevance
**GFAP**/IHC	Positive	Indicates astrocytic (glial) origin of tumor.
**Olig2**/IHC	Positive	Supports glial (astrocytic/oligodendroglial) differentiation.
**Vimentin**/IHC	Positive	Associated with mesenchymal features and tumor aggressiveness.
**MAP2**/IHC	Positive	Suggests partial neuronal differentiation or tumor plasticity.
**p53**/IHC	Positive	Suggests TP53 gene alteration, common in high-grade gliomas.
**H3K27me3**/IHC	Retained	Rules out diffuse midline glioma (which shows H3K27me3 loss).
**IDH-1 R132H**/IHC	Negative	Supports diagnosis of IDH wild-type glioblastoma.
**CDKN2A**/IHC	Negative	No evidence of homozygous deletion; important for molecular grading.
**Synaptophysin**/IHC	Negative	Helps exclude neuronal/neuroendocrine tumors.
**ATRX**/IHC	Retained	Consistent with IDH wild-type glioblastoma (ATRX loss often linked to IDH-mutant astrocytomas).
**Ki-67**/IHC	30%	High proliferation index, typical for glioblastoma, WHO grade IV.
**IDH1/IDH2 Mutation**/PCR	No mutations detected	Molecular confirmation of IDH wild-type status.
**1p/19q Codeletion**/FISH	Loss of 19q13 only	Not compatible with oligodendroglioma; supports glioblastoma diagnosis.

[1] All analyzes were carried out by the hospital's pathology department as part of routine diagnostics.

RNA Extraction, cDNA Synthesis, and Real-Time Polymerase Chain Reaction

Tumor and peritumor tissues collected during surgery were preserved in RNAlater® RNA Stabilization Solution (Ambion™, Applied Biosystems by Thermo Fisher Scientific, Waltham, MA, USA). Total RNA was extracted by homogenizing 100 mg of tissue in 1 ml of TRIzol™ Reagent (Thermo Fisher Scientific), followed by the addition of 0.2 ml chloroform. After vortexing, samples were centrifuged at 12,000 × g for 15 minutes at 4 °C. The aqueous phase containing RNA was transferred to a clean tube, mixed with 0.5 ml of isopropanol, and frozen at −80 °C overnight.

The following day, samples were centrifuged again (12,000 × g, 4 °C, 10 minutes), and the resulting RNA pellets were washed with 75% ethanol. After a final centrifugation step (7,500 × g, 4 °C, 5 minutes), the pellets were air-dried and resuspended in 100 µl of UltraPure™ Water (Gibco, Thermo Fisher Scientific). RNA concentration and purity were assessed spectrophotometrically at 260 nm using a Nanophotometer® N60 (IMPLEN, Munich, Germany). The ratios of OD260/OD280 and OD260/OD230 were used as indicators of RNA quality.

Subsequently, cDNA was synthesized from total RNA using the High Capacity cDNA Reverse Transcription Kit (Applied Biosystems by Thermo Fisher Scientific). Quantitative real-time PCR (qPCR) was performed using SYBR™ Green reagents on a QuantStudio™ 3 Real-Time PCR System (Applied

Biosystems by Thermo Fisher Scientific). Target gene expression levels were quantified using the comparative 2^−ΔCt method, with hypoxanthine phosphoribosyltransferase 1 (*HPRT1*) serving as the endogenous reference gene. Primer sequences for target genes are provided in Supplementary Table 2. Each sample was analyzed in at least two technical replicates. Results are reported as the relative expression of target genes in tumor tissue compared to corresponding gene in peritumor tissue.

Supplementary Table S2. Primer sequences used for qPCR analysis.

Primer	Sequence	Accession No.
HPRT1	f: CCTGGCGTCGTGATTAGTGAT<br>r: AGACGTTCAGTCCTGTCCATAA	NM_000194.3
GFAP	f: CAGGTCCATGTGGAGCTTGAC<br>r: GCCATTGCCTCATACTGCGT	NM_002055.5
VIM	f: AAATGGCTCGTCACCTTCGT<br>r: AGAAATCCTGCTCTCCTCGC	NM_003380.5
SPP1	f: GAAGTTTCGCAGACCTGACAT<br>r: GTATGCACCATTCAACTCCTCG	NM_000582.3
TSPO	f: CCTGCTCTACCCCTACCTGG<br>r: GCCATACGCAGTAGTTGAGTG	NM_000714.6
AR	f: GGAGGCGACAGAGGGAAAAA<br>r: TTCACCGAAGAGGAAAGGGC	NM_000044.6
CYP19A1	f: TGGAAATGCTGAACCCGATAC<br>r: AATTCCCATGCAGTAGCCAGG	NM_001347252.2
ESR1	f: GGGAAGTATGGCTATGGAATCTG<br>r: TGGCTGGACACATATAGTCGTT	NM_001122742.2
GPER1	f: GCTTTCTCGGGGGAGACCTTC<br>r: TGAACCTCACATCCGACTGC	NM_001505.3
PGR	f: ATCGGGGTAAGCCTTGTTGT<br>r: GAAGGGTCGGACTTCTGCTG	NM_000926.4
